# Tuberculosis Stewardship: Reducing Diagnostic Delay Across the Clinical Spectrum in Low-Incidence Settings

**DOI:** 10.3390/microorganisms14020318

**Published:** 2026-01-29

**Authors:** Sara Benevento, Niccolò Riccardi, Giovanni Fumagalli, Luigi Ruffo Codecasa, Giovanni Sotgiu

**Affiliations:** 1 IRCCS Ca’ Granda Ospedale Maggiore di Milano, 20122 Milan, Italy; 2 TB Reference Center and Laboratory, ASST Grande Ospedale Metropolitano Niguarda, 20159 Milan, Italy; 3 Clinical Epidemiology and Medical Statistics Unit, Department of Medicine, Surgery and Pharmacy, University of Sassari, 07100 Sassari, Italy

**Keywords:** tuberculosis, stewardship, infection, screening, treatment, delay

## Abstract

Tuberculosis (TB) remains a leading cause of infectious-disease-related morbidity and mortality worldwide, including in low-incidence, high-income countries, where cases increasingly cluster among vulnerable populations. In these settings, persistent diagnostic and treatment delays, rather than a lack of therapeutic options, drive preventable morbidity, ongoing transmission, and inappropriate antimicrobial use. We argue that TB antimicrobial stewardship must extend beyond treatment adherence and resistance containment to encompass the entire diagnostic continuum. Emerging evidence demonstrating a substantial burden of subclinical and asymptomatic TB challenges symptom-based diagnostic paradigms and reveals an underrecognized “asymptomatic delay”, during which radiologic or microbiologic disease is present but undetected. Failure to identify TB during this interval represents a critical stewardship failure, perpetuating empirical broad-spectrum antibiotic exposure while allowing disease progression and transmission. We review diagnostic challenges across the early clinical spectrum of pulmonary and extrapulmonary TB in low-incidence settings, with particular emphasis on migrants and other high-risk populations disproportionately affected by structural and healthcare system barriers. We propose a stewardship-oriented framework integrating targeted screening, enhanced clinical vigilance, front-loaded and parallel diagnostic pathways, and early referral to specialized TB centers. Explicit incorporation of asymptomatic delay into TB diagnostic frameworks can strengthen system accountability, reduce inappropriate antibiotic use, improve patient outcomes, and accelerate progress toward TB elimination in high-income, low-incidence countries.

## 1. Introduction

Tuberculosis (TB) remains the leading causes of infectious-disease-related morbidity and mortality worldwide, despite the availability of effective diagnostics, curative treatment, and decades of public health experience [1]. In low-incidence, high-income countries, TB increasingly concentrates among vulnerable and marginalized populations, including migrants from high-incidence regions, people experiencing homelessness, individuals living with human immunodeficiency virus (HIV), and those with substance use disorders [1]. In these settings, TB elimination efforts are less constrained by drug availability than by failures in timely diagnosis, and linkage to care. Delays in diagnosis and treatment continue to significantly contribute to preventable morbidity, sustained disease transmission, unnecessary healthcare costs, and the emergence of drug resistance [1,2,3]. Within this context, antimicrobial stewardship (AMS) in TB must be conceptualized beyond the traditional focus on treatment adherence and resistance containment. TB stewardship encompasses the entire care continuum, from appropriate diagnostic suspicion and rational use of microbiological testing to early initiation of effective therapy [1,2,3]. Delayed or missed diagnosis represents a form of stewardship failure: patients are often exposed to repeated courses of inappropriate broad-spectrum antibiotics, while *Mycobacterium tuberculosis* continues to replicate unchecked, increasing transmission risk and disease severity. Diagnostic uncertainty, fragmented care pathways, and limited system-level accountability further exacerbate these challenges. Recent evidence demonstrating a substantial burden of subclinical and asymptomatic TB has fundamentally reshaped the understanding of TB natural history [3,4]. These findings challenge symptom-based diagnostic paradigms and underscore the importance of pro-active, targeted detection strategies as a core component of TB stewardship. In low-incidence countries, where universal screening is neither feasible nor cost-effective, optimizing early detection among high-risk populations is essential to reduce diagnostic delay, improve patient outcomes, and advance TB elimination goals. This manuscript examines diagnostic delays across the early clinical spectrum of TB in low-incidence, high-income settings, reframes delay through the lens of asymptomatic disease, and proposes a stewardship-oriented approach that integrates targeted screening, enhanced clinical vigilance, and streamlined diagnostic pathways, in order to facilitate patients’ referral to reference TB centers, ultimately providing timely and effective care.

## 2. Materials and Methods

On 21 December 2025, we performed a MEDLINE/PubMed search. The complete search string was as follows: ((tuberculosis) AND (stewardship)) AND ((“1 January 2000” [Date—Publication]: “12 December 2025” [Date—Publication])). Of the 208 papers identified, 199 were excluded by title/abstract screening. The full-text of the remaining 9 papers and of their pertinent references was then reviewed and discussed, and the final decision about which papers consider for inclusion in the present paper was made upon the subjective impression of the authors. Table 1 highlights the 9 papers retrieved from the MEDLINE/PubMed search [2,3,5,6,7,8,9,10]. The text was ultimately organized in the following major paragraphs: (i) “Diagnostic Challenges in the Early Clinical Spectrum of TB”; (ii) “Reframing Delay: The Asymptomatic Interval as a Stewardship Challenge”; (iii)” Improving Clinical Recognition and Diagnostic Pathways”; (iv) “Proposed Stewardship-Oriented Solutions”.

## 3. Results and Discussion

### 3.1. Diagnostic Challenges in the Early Clinical Spectrum of TB

Both early pulmonary and extrapulmonary TB frequently present with nonspecific or mild clinical manifestations. Cough, low-grade fever, fatigue, night sweats, and subtle extrapulmonary symptoms are often attributed by patients to self-limiting conditions, resulting in delayed care-seeking and so-called “patient delay” [2]. These missed opportunities for early medical evaluation are particularly relevant in light of robust evidence demonstrating that a substantial proportion of individuals with TB harbor radiological abnormalities in the absence of overt symptoms [3]. Such subclinical or asymptomatic TB can contribute meaningfully to community transmission, challenging long-standing assumptions that infectiousness is confined to symptomatic disease [4]. Diagnosis during the asymptomatic or minimally symptomatic phase confers clear clinical and public health advantages. Early treatment initiation can prevent progression to advanced disease, reduce the risk of severe complications, and interrupt transmission chains. Consequently, targeted screening of groups at elevated risk of TB infection and disease represents a cornerstone of early detection and a critical stewardship intervention [12]. Migrants recently arriving from high-TB-incidence countries are disproportionately affected. In addition to a higher baseline risk of TB infection and reactivation, these individuals often experience prolonged diagnostic delays due to structural and social barriers, including limited access to primary care, administrative and legal obstacles, language barriers, fear of stigmatization, and unfamiliarity with local healthcare systems [13,14]. These delays not only worsen individual outcomes but also facilitate ongoing transmission within vulnerable communities. Data from a large screening initiative conducted in Milan (Italy) illustrate the magnitude of undetected disease in urban, low-incidence settings. Among foreign-born individuals and asylum seekers screened, 68% had evidence of TB infection, and 0.6% had active TB, corresponding to an incidence of 600 cases per 100,000 screened individuals, which is higher than background population rates [14,15]. These findings support the implementation of structured, proactive, and repeated screening strategies in populations at risk as a key pillar of TB elimination efforts.

### 3.2. Reframing Delay: The Asymptomatic Interval as a Stewardship Challenge

Traditional frameworks for TB delay distinguish between patient delay and healthcare system delay. However, emerging evidence necessitates the recognition of an additional interval: the asymptomatic delay, defined as the period between the onset of radiologic or microbiologic evidence of TB and the development of symptoms sufficient to prompt care-seeking [3]. Unlike patient delay, asymptomatic delay is predominantly driven by health system capacity, screening policies, and diagnostic paradigms. Failure to identify TB during this interval represents a missed stewardship opportunity. From an AMS perspective, asymptomatic delay reflects suboptimal allocation of diagnostic resources and perpetuates reliance on empirical, non-specific antibiotic treatments that neither benefit the patient nor control TB transmission. Incorporating the asymptomatic interval into modern TB delay frameworks could enhance system-level accountability, promote earlier detection strategies, and align TB control more closely with stewardship principles.

### 3.3. Improving Clinical Recognition and Diagnostic Pathways

Reducing diagnostic delay requires sustained clinical vigilance across all levels of care. TB remains a “great imitator,” with presentations ranging from classic pulmonary disease to atypical or organ-specific manifestations involving lymph nodes, pleura, bone, central nervous system, or genitourinary tract [16,17]. These presentations can closely resemble malignancies, autoimmune disorders, or other chronic infections, frequently leading to diagnostic misdirection. Primary care physicians, emergency department clinicians, and outpatient specialists play a pivotal role in early TB recognition. Persistent respiratory symptoms, unexplained fever or weight loss, lymphadenopathy, or atypical extrapulmonary findings should prompt TB consideration, particularly in individuals with risk factors such as recent migration from high-incidence countries, homelessness, incarceration, HIV infection, malnutrition, and/or on immunosuppressive treatment [18,19]. Radiological expertise is critical. While classic imaging features, upper lobe infiltrates, cavitation, tree-in-bud nodules, substantially increase pre-test probability, non-specific or subtle abnormalities should not be dismissed in at-risk individuals [20]. Early and parallel microbiological evaluation, including smear microscopy, culture, and molecular testing, should be prioritized [21]. In extrapulmonary disease, early tissue sampling for microbiology and histopathology is essential, particularly given TB’s capacity to mimic neoplastic processes [22]. Immunological assays for TB infection (TBI), including the tuberculin skin test (TST), interferon-gamma release assays (IGRAs), and novel antigen-specific skin tests such as Diaskintest, constitute key components of contemporary screening algorithms for TBI, particularly in the context of targeted TB preventive treatment (TPT). These tests measure host adaptive immune responses to *Mycobacterium tuberculosis*-specific antigens, reflecting immunological sensitization following exposure [23]. Despite their established role in TBI screening, immunological tests have inherent biological and methodological limitations that preclude their application as diagnostic tools for active TB. Critically, these assays do not provide information on mycobacterial viability or disease activity and are unable to discriminate between infection, subclinical disease, and active TB, as all states may elicit comparable antigen-specific T-cell responses. In addition, immunological test performance may be affected by host factors such as immune suppression, age, and recent exposure. Systematic reviews and international guidelines consistently conclude that neither TST, IGRAs, nor antigen-specific skin tests have sufficient sensitivity or specificity to confirm or exclude active TB. Consequently, their use in routine diagnostic pathways for active TB is not recommended, and definitive diagnosis should rely on microbiological, molecular, radiological, and clinical assessment within integrated diagnostic algorithms [24]. Sequential, fragmented diagnostic pathways contribute significantly to healthcare system delay. The adoption of comprehensive, front-loaded diagnostic strategies and early referral to specialized TB centers, where multidisciplinary expertise, advanced diagnostics, and coordinated care pathways are available, can markedly shorten time to diagnosis and treatment initiation. Such approaches embody diagnostic stewardship by ensuring that the right tests are performed at the right time for the right patients.

### 3.4. Proposed Stewardship-Oriented Solutions

**Figure 1:**
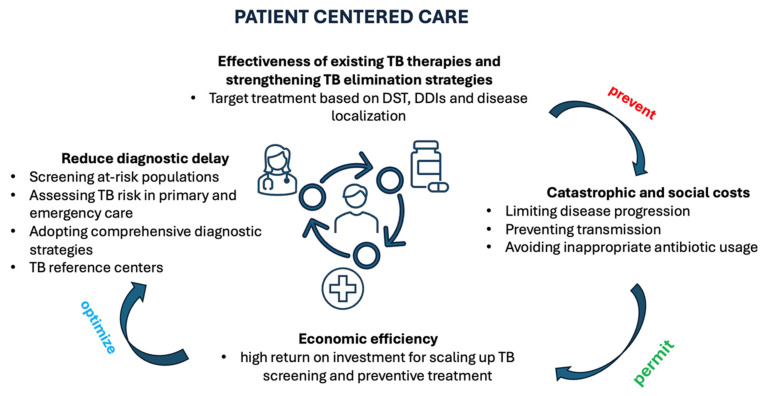
Proposed solutions to decrease delays in TB care. Legend: DDIs = drug–drug interactions; DST = drug susceptibility test; TB = tuberculosis.

Efforts to reduce diagnostic and treatment delays must engage the entire healthcare system and explicitly incorporate antimicrobial stewardship principles (Figure 1). Tailored screening of high-risk populations can identify TB across the disease spectrum, from infection to asymptomatic and active disease [6,25]. Integrating TB risk assessment into routine primary care and emergency department workflows can further expand case detection, particularly among individuals who may not otherwise access preventive services. Of equal importance are interventions addressing social determinants of health: culturally sensitive communication, multilingual navigation services, and reduced bureaucratic barriers are essential to facilitate timely healthcare access and reduce inequities in diagnosis [26]. From a stewardship perspective, these interventions reduce inappropriate antibiotic use, limit disease progression, and prevent transmission, thereby preserving the effectiveness of existing TB therapies and strengthening TB elimination strategies.

## 4. Conclusions

In low-incidence, high-income countries, TB persists not because of a lack of effective tools, but because of delays in their deployment. Targeted screening of high-risk populations, heightened clinical vigilance, streamlined and parallel diagnostic pathways, and early referral to specialized TB services can substantially reduce morbidity, mortality, and community transmission [27]. Although proactive TB screening and health system redesign require initial investment, a substantial and growing evidence base demonstrates that, when appropriately targeted to vulnerable and high-risk populations and delivered by specialized multidisciplinary teams, such approaches are likely to be cost-effective and health-impactful. Systematic economic evaluations indicate that TB screening among high-prevalence and structurally vulnerable groups—such as people living with HIV, recent contacts of infectious cases, migrants from high-incidence regions, individuals experiencing homelessness, and incarcerated populations—can yield favorable cost-effectiveness ratios (e.g., costs per disability-adjusted life year [DALY] averted within acceptable thresholds) compared with passive case finding alone [5,28,29], particularly when coupled with lower-cost initial screening tools followed by molecular diagnostics like Xpert^®^ assays in positive cases.

Health system redesign strategies that integrate TB screening and preventive services into community and primary care, supported by multidisciplinary teams including clinicians, public health practitioners, and social care workers, improve linkage to care, facilitate earlier treatment initiation, and reduce losses along the care cascade—factors shown to enhance both clinical outcomes and economic efficiency. Moreover, recent modelling and investment analyses by the World Health Organization underscore the high return on investment for scaling up TB screening and preventive treatment, with potential economic and health gains far exceeding initial program costs when implemented within comprehensive, risk-stratified frameworks.

By preventing progression to active disease, lowering transmission, reducing catastrophic patient costs, and diminishing inappropriate empirical antimicrobial use, proactive screening and redesigned care pathways align with both public health and health economic goals. Explicit recognition of asymptomatic delay within diagnostic frameworks, coupled with strengthened cross-disciplinary coordination, is essential to modern TB stewardship.

## Figures and Tables

**Table 1:** The nine papers retrieved from the MEDLINE/PubMed search.

	Year	Title	Authors	Journal	Reference
1	2012	Commentary on: Does empirical treatment of community-acquired pneumonia with fluoroquinolones delay tuberculosis treatment and result in fluoroquinolone resistance in *Mycobacterium tuberculosis*? Controversies and solutions	Hara G.L.	*International Journal of Antimicrobial Agents*	[5]
2	2016	Antibiotic stewardship for drug resistant tuberculosis	Padayatchi N. et al.	*Expert Opinion on Pharmacotherapy*	[6]
3	2018	Anti-tuberculosis treatment stewardship in a private tertiary care hospital in South India	Prabhu B.P. et al.	*Public Health Action*	[7]
4	2021	Next-Generation Digital Biomarkers for Tuberculosis and Antibiotic Stewardship: Perspective on Novel Molecular Digital Biomarkers in Sweat, Saliva, and Exhaled Breath.	Brasier N. et al.	*Journal Of Medical Internet Research*	[8]
5	2023	Local government stewardship for TB elimination in Kerala, India	Rakesh P.S. et al.	*Public Health Action*	[9]
6	2024	Cycles of antibiotic use and emergent antimicrobial resistance in the South African tuberculosis programme (1950–2021): A scoping review and critical reflections on stewardship	Raad R. et al.	*Glob Public Health*	[10]
7	2025	Why don’t we talk about tuberculosis stewardship?	Riccardi N. et al.	*Clinical Microbiology and Infection*	[2]
8	2024	Adopting a model of antimicrobial stewardship program to anti-tubercular treatment stewardship: A single-centre experience from a private tertiary care hospital in South India.	Samban S.S. et al.	*Public Library of Science*	[11]
9	2025	Clinical standards for antimicrobial stewardship in TB care	Brehm T.T. et al.	*International Journal of Tuberculosis and Lung Disease Open*	[3]

## Data Availability

The original contributions presented in this study are included in the article. Further inquiries can be directed to the corresponding author.
